# High-fat and low-fat fermented milk and cheese intake, proteomic signatures, and risk of all-cause and cause-specific mortality

**DOI:** 10.1007/s00394-025-03815-6

**Published:** 2025-10-15

**Authors:** Yufeng Du, Ruikun Bao, Shunming Zhang, Ulrika Ericson, Yan Borné, Lu Qi, Emily Sonestedt

**Affiliations:** 1 Department of Epidemiology and Statistics, School of Public Health, Lanzhou University, Lanzhou, Gansu China; 2 Nutritional Epidemiology, Department of Clinical Sciences Malmö, Lund University, Jan Waldenströms gata 35, Malmö, 21428 Sweden; 3 School of Public Health, Xi’an Jiaotong University Health Science Center, Xi’an, Shaanxi China; 4 Diabetes and Cardiovascular Disease-Genetic Epidemiology, Department of Clinical Sciences Malmö, Lund University, Malmö, Sweden; 5 Department of Epidemiology, School of Public Health and Tropical Medicine, Tulane University, New Orleans, LA USA; 6 Department of Nutrition, Harvard T.H. Chan School of Public Health, Boston, MA USA; 7 Department of Food and Meal Science and the Research Environment MEAL, Faculty of Natural Science, Kristianstad University, Kristianstad, Sweden

**Keywords:** Fat, Cheese, Fermented milk, Prospective cohort, Proteins, Mortality

## Abstract

**Purpose:**

This study aimed to examine the associations between the intake of high- and low-fat fermented dairy (cheese and fermented milk), their proteomic profiles, and mortality risk.

**Methods:**

This cohort study included 25,187 participants (mean age 57.7 years, 60.9% females). Fermented dairy intake was assessed by a modified diet history method. In a random subset of this cohort (*n* = 4359), we constructed proteomic signatures for fermented dairy intake using 136 candidate plasma proteins.

**Results:**

During 23.5 years of follow-up, 9742 participants died. High-fat cheese (> 20% fat) intake was inversely associated with risk of all-cause mortality (HR for an increment of 20 g/day, 0.97; 95% CI, 0.96–0.99, *P* < 0.001) and cardiovascular disease mortality (HR, 0.96; 95% CI, 0.93–0.99, *P* = 0.006). Low-fat cheese intake showed an inverse association with all-cause mortality (HR, 0.98; 95% CI, 0.96–1.00, *P* = 0.047). Low-fat fermented milk intake was inversely associated with all-cause mortality (HR for an increment of 250 g/day, 0.91; 95% CI, 0.85–0.97, *P* = 0.006), while high-fat fermented milk (> 2.5% fat) showed null association. A total of 42, 26, 0, and 39 proteins were identified for the signature of high-fat cheese, low-fat cheese, high-fat fermented milk, and low-fat fermented milk, respectively. Inverse associations with all-cause mortality were observed for all three signatures with identified proteins. The identified proteins were involved in biological pathways related to immune response and inflammation.

**Conclusion:**

Our study indicated that consuming high-fat cheese, low-fat cheese, and low-fat fermented milk was linked to survival benefits. Plasma proteins improve our understanding of the health effects of fermented dairy.

**Supplementary Information:**

The online version contains supplementary material available at 10.1007/s00394-025-03815-6.

## Introduction

Dairy foods constitute important parts of traditional Western diets, and consumption of low-fat, rather than high-fat, dairy foods is recommended in dietary guidelines across many countries [1]. This emphasis on low-fat dairy is mainly due to concerns about saturated fats and high calories. However, controversy continues regarding the consumption of high-fat or low-fat dairy, and growing evidence, including that from randomized controlled trials (RCTs), showed neutral or possibly beneficial cardiometabolic health effects of high-fat dairy [2–5]. RCTs typically had short intervention periods and encountered difficulties in observing long-term health effects, particularly for mortality events. Nevertheless, prior prospective cohort studies [6, 7], including ours [8], with long-term follow-up and real-life dietary data, generally did not differentiate the fat content of fermented dairy in their analyses, even though such differentiation has long been expected. In addition, intake of dairy products in Sweden is among the highest in the world, uniquely enabling us to investigate the health effects of extremely high consumption in the Sweden populations.

Proteomics, which analyzes large sets of proteins from a single biospecimen, may shed light on physiological responses to diet, the molecular mechanisms underlying diet and health conditions, and biomarker-driven nutritional interventions [9]. Dietary interventions, such as high-protein diet and Mediterranean diet, have been shown to significantly affect the plasma proteome [10, 11]. Prior cohort studies have identified the proteomic signatures of various dietary exposures, such as plant-based diet, inflammatory diet, milk, and diet patterns (e.g., Mediterranean diet) [9, 12–18], and linked them to disease risk [13, 14, 18]. To our knowledge, no cohort studies have utilized proteomics data to assess the associations between proteomic signatures of high- and low-fat fermented dairy intake and future mortality risk.

We aimed to investigate the association of high- and low-fat fermented dairy intake with all-cause and cause-specific mortality in the Malmö Diet and Cancer (MDC) cohort. We also identified plasma proteomic signatures for fermented dairy intake and assessed their associations with all-cause mortality.

## Methods

### Study population

The MDC is a prospective cohort conducted in Malmö, Sweden between 1991 and 1996, with adults aged 45–73 years enrolled. The study invited all Malmö residents born 1923–1950 for women and 1923–1945 for men to participate (*n* = 74,318). Individuals with insufficient Swedish language skills and mental disabilities that impeded completion of the baseline questionnaire were excluded [19]. Participants were asked to visit the study center twice. At the first visit, a set of self-administered questionnaires assessing lifestyle, sociodemographic factors, and diet were explained and distributed to participants. Participants received anthropometric measurements and provided blood samples. Approximately two weeks later, participants returned their questionnaires, and a diet interview was conducted. More details of study design have been described previously [19, 20]. The study was approved by the Ethical Committee at the Medical Faculty at Lund University (approval number: LU 51/90). Written informed consent was obtained from all participants.

Of the 30,446 participants who took part in the baseline examination, 28,098 individuals completed the questionnaire, anthropometric measurements, and dietary assessment. We excluded those with missing data on 7 covariates (*n* = 428) and those with prevalent cancer or cardiovascular disease (CVD) (*n* = 2483), finally leaving 25,187 participants in the present analyses.

Between 1991 and 1994, a random subset of participants from the MDC was invited to participate in the Malmö Diet and Cancer Cardiovascular Cohort (MDC-CC). The MDC-CC included 6103 participants, with 5543 of them providing blood samples following standardized overnight fasting. Those with prevalent cancer or CVD, or with missing covariates and proteomic data, were further excluded. The final analysis involved 4359 participants for proteins. The flowchart (Supplementary Fig. 1) provides more details.

### Dietary assessment

In MDC, diet was assessed using a modified diet history method which combined a 7-day food diary, a 168-item semiquantitative food frequency questionnaire (FFQ), and a 45–60 min dietary interview. This method was validated by a reference method of 18-day weighted food records and showed good validity and reproducibility [21, 22]. Participants recorded their daily meals (usually cooked lunch and dinner), cold beverages, and dietary supplements for 7 consecutive days in the food diary. The food diary collected information on dairy use in cooked meals. FFQ was utilized to assess the intake of regularly consumed foods over the past year, mainly breakfast, hot beverages, and snacks. The FFQ included questions about the intake of cheese on bread, other cheese (e.g., cheese plates), yogurt, and other fermented milks (e.g., sour milk). In addition, a dietary interview was carried out to quantify the food amounts and collect details of food preparation in the food diary, as well as verify the overlap of foods between the FFQ and the food diary. The average daily intake for each food item (g/day) was calculated by summarizing the food data from the food diary and FFQ. Total energy and nutrient intake were calculated using computer software and the Swedish Food Database PC KOST2-93 of the Swedish National Food Administration.

The fat thresholds that differentiate low- from high-fat fermented dairy were 2.5% for fermented milk and 20% for cheese. The selection of these thresholds was based on the original items in the FFQ. Our threshold for high-fat cheese matched previous studies [23, 24], but was slightly higher for high-fat fermented milk (2.5% vs. 2%). We categorized cheese and fermented milk intake into four categories. The distribution of intake was the primary consideration in classification.

### Measurement of plasma proteins

Overnight fasting blood samples were collected, centrifuged to separate plasma, and then stored at − 80 °C until analysis. Using the Olink platform (Olink Proteomics, Uppsala, Sweden), 149 plasma proteins were quantified. The Olink technique employs proximity extension assay (PEA) technology, in which two oligonucleotide-labelled antibodies bind to a target protein, forming a PCR target sequence that is quantified by real-time quantitative polymerase chain reaction, demonstrating high specificity and sensitivity [25, 26]. Thirteen proteins were excluded due to the proportion of missing value >25%, leaving 136 proteins in the analysis. The proportions of missing values for 136 proteins are as follows: 106 have < 3%, 24 have 3–9%, and 6 have 10–24%. For these proteins, the random forest imputation method was employed to fill in missing values [27]. Protein concentrations were expressed as relative quantification units on a log2 scale and standardized (mean = 0, standard deviation = 1) for further analysis.

### Outcome ascertainment

Deaths and emigrations were ascertained by linking to the Swedish National Tax Agency, Statistics in Sweden, and the National Board of Health and Welfare, and cause-specific deaths were ascertained by the Swedish Cause of Death Register. Cause-specific deaths were classified using the following codes from the ninth and tenth revisions of the International Classification of Diseases (ICD): 140–239 (ICD-9) and C, D00-D48 (ICD-10) for cancer death, 390–459 (ICD9) and I (ICD-10) for CVD death. A total of 0.8% of participants were lost to follow-up due to emigration.

### Assessment of covariates and other variables

Age and sex data were obtained from the Swedish registry through personal identification number. BMI (kg/m^2^) was calculated based on measured weight and height and further categorized into four groups (< 18.5, 18.5–24.9, 25–29.9, and ≥ 30 kg/m^2^). Data on smoking habits (current, former, or never), educational level (elementary, primary and secondary, upper secondary, further education without a degree, and university degree), marital status (married or others), and whether living alone (yes or no) were obtained from a self-administered questionnaire. We created a 6-category variable for alcohol consumption (zero consumers, and sex-specific quintiles for participants reporting drinking) [28]. Metabolic equivalent task (MET) hours per week were derived from 17 different leisure-time physical activities and were further grouped into five categories (< 7.5, 7.5–15, 15–25, 25–50, and >50 MET-hour/week). The heredity score for cancer or CVD was generated based on self-reported family history, assigning one point if a participant’s relative (father, mother, or siblings) had the corresponding disease. The diet quality index, based on the Swedish dietary guidelines, was established by aggregating five dietary factors: fiber (>2.4 g/MJ), fruit and vegetables (>400 g/day), fish (>300 g/week), added sugar (< 10% energy), and red and processed meat (< 500 g/week) [29]. Each dietary factor is assigned a score of 1 if the above criterion is met, otherwise, the score is 0, resulting in an overall diet score ranging from 0 to 5. The variable dietary assessment method (old or new) was established as a result of shortening dietary interview duration from 60 min to 45 min in 1994. The season (spring, summer, autumn, and winter) of collecting dietary data was also considered as a covariate.

Participants were considered potential energy misreporters if their ratio of energy intake to basal metabolic rate fell outside the 95% CI of the calculated physical activity level [30]. Participants rated their current health status according to the question: “How do you feel right now, physically and mentally, with respect to your health and your well-being?”, with the answer was a score ranging from 1 (feel very bad, could not feel worse) to 7 (feel very well, could not feel better). Participants were considered diet changers if they reported “yes” to the question in the baseline questionnaire: “Have you substantially changed your eating habits because of illness or some other reasons?”. In the five-year follow-up examination (1997–2001), we assessed dietary change status using the question: “Have you substantially changed your dietary habits since you participated in the MDC study for the first time?”.

### Statistical analyses

#### Analysis 1 (fermented dairy intake and mortality risk)

Participants were followed from the baseline until either death, emigration, or December 31, 2018, whichever came first. We used Cox proportional hazards regression models to calculate HRs (95% CIs) for mortality according to fermented dairy intake in categorical and continuous scales. P for trend was calculated by entering the median of each category as a continuous scale in Cox models. We established two models by progressively adjusting covariates. In model 1, age, sex, dietary assessment method, and season were adjusted. Model 2 was additionally adjusted for educational level, leisure-time physical activity, smoking status, alcohol consumption, total energy intake, heredity score of cancer (0 or > 0) or CVD (0 or > 0), marital status, whether living alone, BMI, diet quality index, hypertension, diabetes, lipid-lowering medication, and mutual adjustment for high- and low-fat fermented dairy. We tested the proportional hazards assumption of the Cox model through Schoenfeld test. No violation of this assumption was found. The dose-response associations between fermented dairy intake and mortality were investigated using restricted cubic splines models with 3 knots. Spearman correlations were calculated for dairy foods, other foods, and nutrients.

We did stratified analyses by sex, age, BMI, smoking habits, drinking status, physical activity, diet quality (< 2/≥ 2), and self-rated health condition (< 6/≥ 6). A stratified analysis by self-rated health condition was conducted to evaluate the impact of potential reverse causation due to comorbidity-related changes in dairy intake. Potential interactions were evaluated using the Wald test for cross-product terms (dairy intake categories × stratification variables).

Several sensitivity analyses were performed. First, to assess the impact of potential reverse causality, the following exclusions were made: diet changers at baseline (*n* = 5845), deaths occurring within the first 5 years of follow-up (*n* = 610), incident CVD, cancer, or diabetes cases within the first 5 years of follow-up (*n* = 2535). Second, energy misreporters were excluded (*n* = 4693). Last, we restricted our study sample to those who reported not altering their diet during the five-year follow-up examination (*n* = 16764).

#### Analysis 2 (fermented dairy intake, plasma proteins, and all-cause mortality risk)

Due to the high dimensionality and collinearity of the proteomics data, an elastic net regression model was used for selecting proteins related to dairy intake. The hyperparameters α and λ were determined through 10-fold cross-validation (R package: “caret”). The data were randomly divided into a training set (70%) and a testing set (30%). The elastic net regression models were first fitted in the training set. Subsequently, the coefficients (weights) were used to generate the protein profile score, a weighted sum of the selected proteins, in the testing set. Age and sex were forced into the elastic net regression model without penalty. The protein profile score for the training set was generated using the leave-one-out method to prevent overfitting. These methods have been utilized to identify the diet-related metabolic signatures [31, 32]. Quartiles were generated for each protein profile score, and Cox proportional hazards regression models were used to estimate their associations with all-cause mortality, with two sets of progressive adjustments performed. Model 1 was adjusted for age and sex. Model 2 was further adjusted for season, educational level, leisure time physical activity, smoking status, alcohol consumption, total energy intake, heredity score of cancer or CVD, marital status, whether living alone, BMI, diet quality index, hypertension, diabetes, and lipid-lowering medication.

The following analyses were performed to create heatmaps to observe whether the identified proteomic signatures were specific to the corresponding dairy intake and to present the associations of each protein with all-cause mortality risk. The partial correlations of proteins with the intake of fermented milk, cheese, butter, vegetables and fruits, meat, and soft drinks were calculated. Age, sex, educational level, leisure time physical activity, smoking status, alcohol consumption, BMI, total energy intake, diet quality index, hypertension, diabetes, and lipid-lowering medication were adjusted in this analysis. The association between each protein and all-cause mortality risk was estimated using Cox proportional hazards regression models, and the FDR was used to control for Type 1 error; the same covariates in above Model 2 of the protein profile score were adjusted.

Network analysis and enrichment analyses were performed for the selected proteins in each signature to describe protein interactions and identify potential biological pathways, using the Web-based tool STRING (version 12.0) (https://string-db.org/). The nodes in the network were linked based on all types of interaction evidence, and edges with interaction scores greater than 0.4 are presented. The Kyoto Encyclopedia of Genes and Genomes (KEGG) functional database was used to annotate proteins to pathways. Pathways with a false discovery rate (FDR)-adjusted P value of less than 0.05 were deemed statistically significant.

Statistical analyses were performed with R version 4.2.1 (R Foundation). All tests were two-sided, and the significance was set at 0.05.

## Results

Table 1 summarizes the baseline characteristics of the 25,187 participants, of whom 60.9% were female, and the average age was 57.7 years (SD 7.6 years). Participants with higher intake of high-fat cheese or high-fat fermented milk tended to be younger, had lower BMI, and were less likely to have a family history of CVD, prevalent diabetes, hypertension, or use lipid-lowering medication. Individuals who consumed more low-fat cheese or low-fat fermented milk were typically female, less likely to be current smokers, more physically active, had better diet quality, but were more likely to have prevalent diabetes and use lipid-lowering medication. Participants with higher education levels tended to consume more of all four types of fermented dairy. Self-rated health scores were similar across categories for all exposures. High intake of high-fat cheese and fermented milk was associated with higher total and saturated fat intake but lower fiber intake, whereas high intake of low-fat cheese and fermented milk showed the opposite trend.

**Table 1:** Characteristics by categories of fermented dairy consumption

Characteristics	Total	High-fat cheese			Low-fat cheese			High-fat fermented milk			Low-fat fermented milk	
		< 15 g	≥ 50 g		0 g	≥ 30 g		0 g	≥ 200 g		0 g	≥ 200 g
Baseline												
Participants, n	25,187	6506	6524		14,877	2769		14,710	1540		16,772	1533
Age, years	57.7 ± 7.6	59.0 ± 7.4	56.1 ± 7.2		58.1 ± 7.6	57.2 ± 7.1		58.1 ± 7.5	57.7 ± 7.6		58.0 ± 7.6	57.5 ± 7.3
Female	15,334 (60.9)	4063 (62.5)	3721 (57.0)		8282 (55.7)	1893 (68.4)		8421 (57.3)	884 (57.4)		9460 (56.4)	932 (60.8)
Married	16,474 (65.4)	4213 (64.8)	4082 (62.6)		9668 (65.0)	1770 (64.0)		9890 (67.3)	909 (59.1)		10,833 (64.6)	1009 (65.8)
Living alone	6077 (24.1)	1721 (26.5)	1610 (24.7)		3617 (24.3)	725 (26.2)		3436 (23.4)	422 (27.4)		4129 (24.6)	390 (25.5)
Body mass index, kg/m^2^	25.7 ± 3.9	26.1 ± 4.1	25.3 ± 3.9		25.6 ± 3.9	25.9 ± 4.1		25.9 ± 4.0	25.0 ± 3.7		25.6 ± 4.0	25.7 ± 3.6
University degree	3678 (14.6)	658 (10.1)	1321 (20.3)		1855 (12.5)	515 (18.6)		1821 (12.4)	340 (22.1)		2227 (13.3)	308 (20.1)
Smoking status												
Current	7180 (28.5)	1822 (28.0)	1962 (30.1)		4647 (31.2)	706 (25.5)		4246 (28.9)	436 (28.3)		5288 (31.5)	293 (19.1)
Past	8377 (33.3)	2110 (32.4)	2265 (34.7)		4878 (32.8)	999 (36.1)		4999 (34.0)	534 (34.7)		5467 (32.6)	619 (40.4)
Never	9630 (38.2)	2574 (39.6)	2297 (35.2)		5352 (36.0)	1064 (38.4)		5465 (37.2)	570 (37.0)		6017 (35.9)	621 (40.5)
Zero-consumers of alcohol	1536 (6.1)	657 (10.1)	274 (4.2)		878 (5.9)	229 (8.3)		990 (6.7)	104 (6.8)		1098 (6.6)	89 (5.8)
High leisure-time physical activity (> 50METh/week)	4072 (16.2)	1087 (16.7)	1065 (16.3)		2343 (15.8)	491 (17.7)		2310 (15.7)	291 (18.9)		2676 (16.0)	290 (18.9)
Heredity score of cancer (> 0)	11,552 (45.9)	2973 (45.7)	2979 (45.7)		6756 (45.4)	1329 (48.0)		6794 (46.2)	711 (46.2)		7652 (45.6)	707 (46.1)
Heredity score of CVD (> 0)	13,305 (52.8)	3520 (54.1)	3330 (51.0)		7760 (52.2)	1527 (55.2)		7795 (53.0)	761 (49.4)		8777 (52.3)	800 (52.2)
Diabetes	1034 (4.1)	419 (6.4)	196 (3.0)		487 (3.3)	214 (7.7)		732 (5.0)	36 (2.3)		644 (3.8)	75 (4.9)
Hypertension	15,205 (60.4)	4212 (64.7)	3597 (55.1)		9116 (61.3)	1630 (58.9)		9212 (62.6)	855 (55.5)		10,271 (61.2)	880 (57.4)
Lipid-lowering medication	608 (2.4)	291 (4.5)	69 (1.1)		278 (1.9)	95 (3.4)		450 (3.1)	20 (1.3)		359 (2.1)	48 (3.1)
Self-rated health score	5.3 ± 1.3	5.3 ± 1.4	5.3 ± 1.3		5.3 ± 1.3	5.2 ± 1.3		5.3 ± 1.3	5.3 ± 1.3		5.3 ± 1.3	5.3 ± 1.3
Diet quality index	1.9 ± 1.3	2.1 ± 1.4	1.9 ± 1.2		1.7 ± 1.2	2.5 ± 1.3		1.9 ± 1.3	2.0 ± 1.3		1.8 ± 1.2	2.3 ± 1.4
Total energy intake, kcal/day	2284.5 ± 656.8	2042.6 ± 598.1	2625.6 ± 702.2		2328.4 ± 676.7	2256.0 ± 621.3		2240.2 ± 659.2	2522.0 ± 672.2		2325.7 ± 675.5	2318.1 ± 642.6
Fat, E%	39.1 ± 6.1	36.2 ± 6.3	41.8 ± 5.5		40.1 ± 6.0	36.6 ± 5.8		38.8 ± 6.4	39.4 ± 5.7		40.0 ± 6.0	35.7 ± 6.0
Saturated fat, E%	16.8 ± 3.9	14.7 ± 3.6	19.0 ± 3.6		17.5 ± 3.9	15.4 ± 3.4		16.4 ± 3.9	17.9 ± 3.7		17.4 ± 3.9	15.0 ± 3.6
Fiber, g/1,000 kcal	9.4 ± 2.8	10.3 ± 3.3	8.6 ± 2.3		8.8 ± 2.5	10.6 ± 3.0		9.4 ± 2.9	9.1 ± 2.6		9.0 ± 2.7	10.4 ± 3.0
Added sugar, E%	10.0 ± 4.3	10.2 ± 4.8	9.4 ± 3.9		10.1 ± 4.4	9.2 ± 4.3		9.8 ± 4.4	10.3 ± 4.5		10.0 ± 4.4	10.2 ± 4.7
Five-year follow-up examination ^b^												
Married	11,568 (64.3)	2899 (64.0)	2945 (61.7)		6701 (64.2)	1285 (62.9)		6924 (66.4)	598 (55.3)		7428 (63.3)	763 (65.3)
Living alone	4766 (26.5)	1277 (28.2)	1294 (27.1)		2759 (26.4)	591 (28.9)		2632 (25.2)	360 (33.3)		3196 (27.2)	321 (27.5)
High leisure-time physical activity (> 50METh/week)	2725 (15.2)	693 (15.3)	730 (15.3)		1516 (14.5)	344 (16.9)		1579 (15.1)	188 (17.4)		1716 (14.6)	228 (19.5)
Smoking status												
Current	3817 (21.2)	921 (20.3)	1105 (23.2)		2451 (23.5)	383 (18.8)		2228 (21.4)	225 (20.8)		2765 (23.6)	172 (14.7)
Past	6945 (38.6)	1746 (38.5)	1890 (39.6)		4042 (38.7)	825 (40.4)		4135 (39.6)	434 (40.1)		4572 (39.0)	485 (41.5)
Never	7227 (40.2)	1864 (41.1)	1775 (37.2)		3948 (37.8)	834 (40.8)		4072 (39.0)	423 (39.1)		4399 (37.5)	511 (43.8)
Self-rated health score	5.4 ± 1.3	5.4 ± 1.3	5.4 ± 1.2		5.4 ± 1.3	5.3 ± 1.3		5.4 ± 1.3	5.3 ± 1.3		5.4 ± 1.3	5.4 ± 1.2

^a^ Variables are presented as mean ± SD or n (%)

^b^ A total of 17,989 participants were included in the five-year follow-up

In addition, we presented several variables to observe changes in participant characteristics over time, collected during the five-year follow-up examination (Table 1). There was an increase in the proportion of individuals living alone and former smokers, but a decrease in high physical activity, whereas the self-rated health score remained largely unchanged. The distribution of these factors across fermented dairy categories was similar to that at baseline.

Supplementary Fig. 2 displays the correlation matrix of various foods and nutrients. Consumption of high-fat cheese was negatively correlated with low-fat cheese (*r* = − 0.27); a similar correlation was observed for the two types of fermented milk (*r* = − 0.23). Intake of high-fat cheese and fermented milk was positively associated with saturated fat and butter, whereas low-fat cheese and fermented milk were positively linked to fiber, whole grains, and fruits and vegetables. The magnitude of the associations of four types of fermented dairy with other foods was generally weak (r range: − 0.16–0.20).

Supplementary Fig. 3 shows the distribution of fermented dairy intake by fat content. The distribution of high-fat and low-fat fermented milk intake were similar. However, our participants consumed more high-fat cheese than low-fat cheese, and a large proportion of them did not consume low-fat cheese.

### Fermented dairy intake and mortality risk

**Fig. 1:**
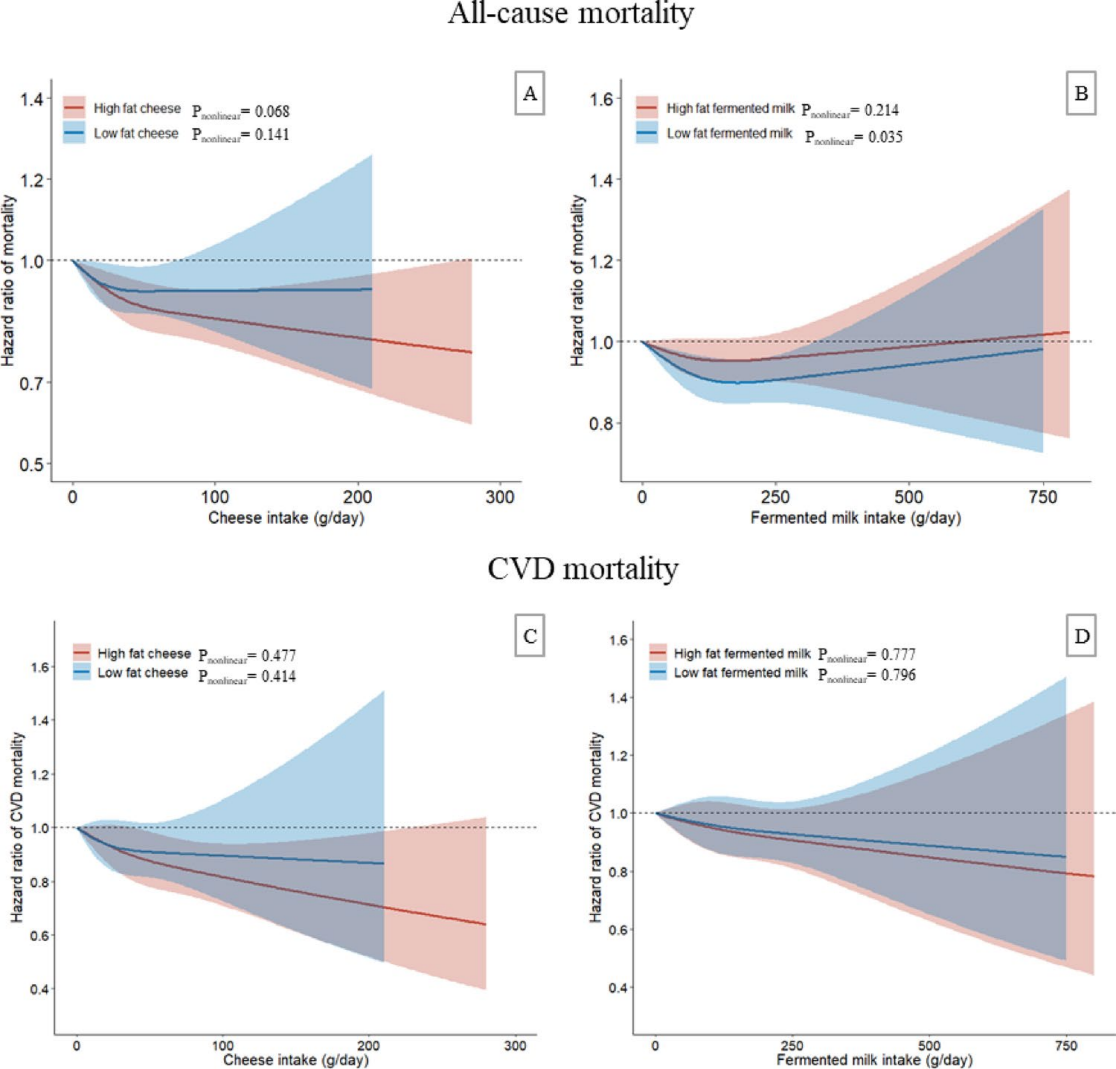
Dose-response associations of all-cause mortality and CVD mortality with cheese (**A** and** C**) or fermented milk (**B** and** D**). No consumption was used as reference level (HR = 1). Covariates in model 2 were adjusted

**Fig. 2:**
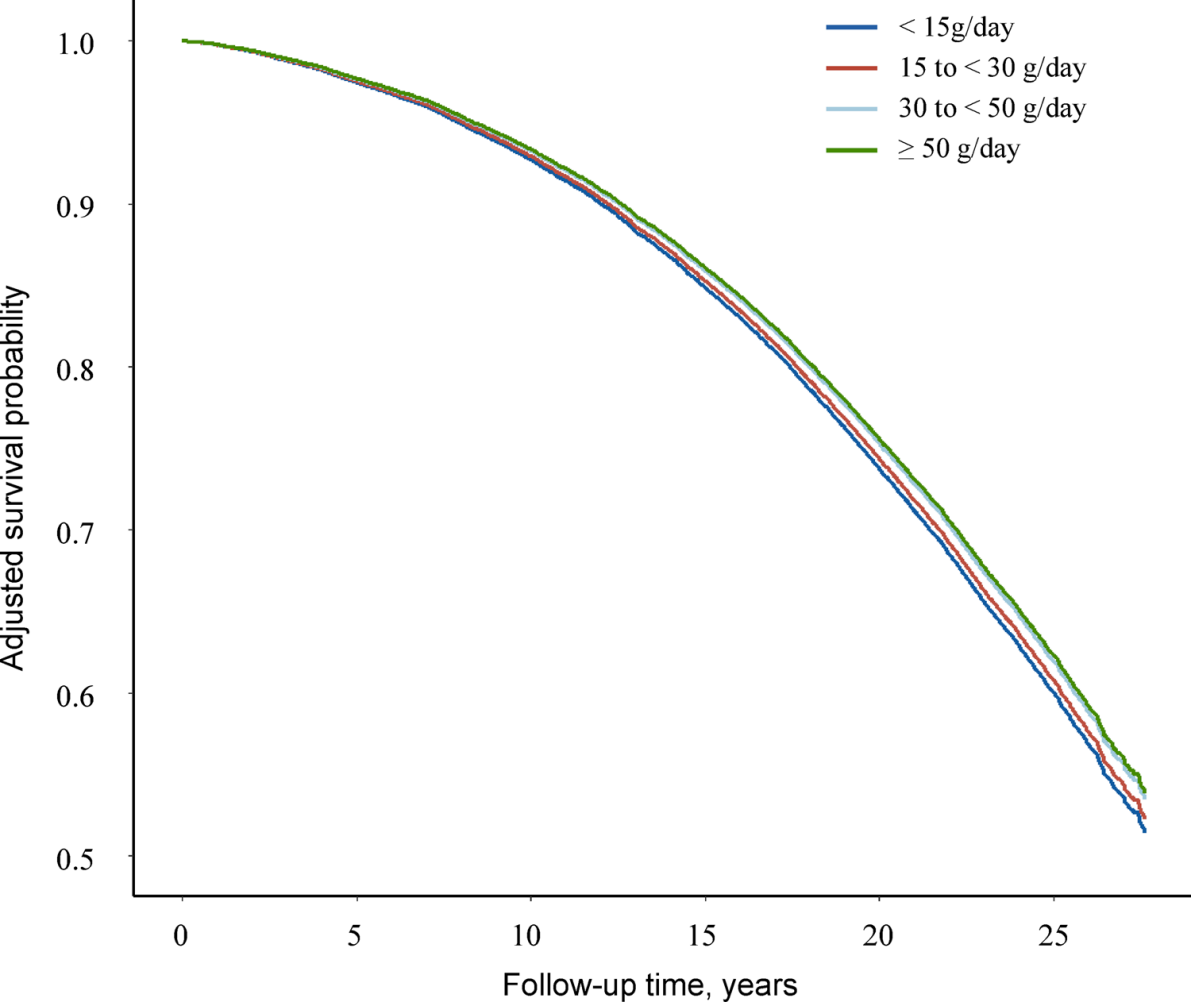
Adjusted survival probability by category of high-fat cheese consumption. Covariates in model 2 were adjusted

After a median follow-up of 23.5 years (539179 person-years), 9742 participants died (of cancer, 3380; of CVD, 3115). The spline analysis revealed linear inverse associations between all-cause mortality and both high- and low-fat cheese, as well as between high-fat cheese and CVD mortality (Fig. 1). As shown in Table 2, for each 20 g/day increase in high-fat cheese intake, the HR (95% CI) was 0.97 (0.96–0.99) for all-cause mortality and 0.96 (0.93–0.99) for CVD mortality. A similar but weaker association was observed for low-fat cheese and all-cause mortality (HR per 20 g/day increase, 0.98; 95% CI, 0.96–1.00, *P* = 0.047). High-fat fermented milk showed no significant associations with all-cause or cancer mortality, but a borderline inverse association was observed with CVD mortality. The association between low-fat fermented milk and all-cause mortality was nonlinear, with the lowest risk observed at approximately 180 g/day (Fig. 1). Participants who consumed 100 to < 200 g/day of low-fat fermented milk (vs. no consumption) had a 12% lower risk of all-cause mortality (HR, 0.88; 95% CI, 0.82–0.95). All fermented dairy was not associated with cancer mortality, except for low-fat fermented milk, which showed a borderline inverse association (P_trend_=0.047). In addition, intake of total cheese or fermented milk showed inverse associations with all-cause mortality (data not shown), similar to findings from our previous study with a shorter follow-up period (19 years) [8]. The adjusted survival probabilities by different levels of high-fat cheese intake are presented in Fig. 2. Greater survival benefits were seen with increasing high-fat cheese intake compared to lower intake.

**Table 2:** Hazard ratios (95% CIs) for all-cause and cause-specific mortality by high- and low-fat fermented dairy consumption^a^

	Categories of fermented dairy consumption				*P*_trend_	Continuous	
**High-fat cheese**	**< 15 g**	**15 to < 30 g/day**	**30 to < 50 g/day**	**≥ 50 g/day**		**Per 20 g increase**	*P*
All-cause mortality							
Cases/person years	2902/136,303	2397/124,654	2316/135,176	2127/143,047	–	–	–
Model 1	Ref.	0.93 (0.88–0.98)	0.88 (0.84–0.93)	0.87 (0.82–0.92)	< 0.001	0.97 (0.95–0.98)	< 0.001
Model 2	Ref.	0.97 (0.92–1.03)	0.93 (0.88–0.98)	0.91 (0.86–0.97)	0.002	0.97 (0.96–0.99)	< 0.001
CVD mortality							
Cases	971	777	718	649	–	–	–
Model 1	Ref.	0.90 (0.82–0.99)	0.83 (0.75–0.91)	0.82 (0.74–0.91)	< 0.001	0.95 (0.92–0.97)	< 0.001
Model 2	Ref.	0.96 (0.87–1.06)	0.90 (0.81–0.99)	0.89 (0.79–0.99)	0.023	0.96 (0.93–0.99)	0.006
Cancer mortality							
Cases	954	824	793	809	–	–	–
Model 1	Ref.	0.97 (0.88–1.07)	0.89 (0.81–0.98)	0.93 (0.84–1.02)	0.063	0.98 (0.96–1.01)	0.166
Model 2	Ref.	0.99 (0.89–1.09)	0.90 (0.82-1.00)	0.94 (0.85–1.05)	0.180	0.99 (0.96–1.01)	0.288
Low-fat cheese	**0 g**	**< 10 g/day**	**10 to < 30 g/day**	**≥ 30 g/day**		**Per 20 g increase**	*P*
All-cause mortality							
Cases/person years	6096/313,802	1384/88,907	1239/76,022	1023/60,448	–	–	–
Model 1	Ref.	0.92 (0.87–0.98)	0.89 (0.84–0.94)	0.98 (0.92–1.05)	0.192	0.98 (0.96-1.00)	0.055
Model 2	Ref.	0.97 (0.91–1.03)	0.92 (0.86–0.98)	0.96 (0.90–1.03)	0.148	0.98 (0.96-1.00)	0.047
CVD mortality							
Cases	1955	427	424	309	–	–	–
Model 1	Ref.	0.93 (0.84–1.03)	0.98 (0.88–1.08)	0.97 (0.86–1.09)	0.660	0.99 (0.95–1.02)	0.492
Model 2	Ref.	0.96 (0.86–1.06)	0.99 (0.89–1.10)	0.91 (0.80–1.03)	0.182	0.97 (0.93–1.01)	0.148
Cancer mortality							
Cases	2133	475	417	355	–	–	–
Model 1	Ref.	0.88 (0.80–0.97)	0.84 (0.76–0.94)	0.94 (0.84–1.05)	0.113	0.97 (0.93-1.00)	0.060
Model 2	Ref.	0.94 (0.85–1.04)	0.91 (0.82–1.01)	0.97 (0.87–1.10)	0.511	0.98 (0.95–1.02)	0.313
High-fat fermented milk	**0 g**	**< 100 g/day**	**100 to < 200 g/day**	**≥ 200 g/day**		**Per 250 g increase**	*P*
All-cause mortality							
Cases/person years	5981/311,722	1964/127,107	1213/67,292	584/33,058	–	–	–
Model 1	Ref.	0.94 (0.89–0.99)	0.93 (0.88–0.99)	0.92 (0.85–1.01)	0.008	0.93 (0.88–0.99)	0.026
Model 2	Ref.	0.96 (0.91–1.01)	0.95 (0.89–1.01)	0.95 (0.87–1.04)	0.095	0.96 (0.91–1.02)	0.236
CVD mortality							
Cases	1959	616	374	166	–	–	–
Model 1	Ref.	0.94 (0.86–1.03)	0.88 (0.79–0.98)	0.79 (0.68–0.93)	0.001	0.85 (0.76–0.95)	0.004
Model 2	Ref.	0.98 (0.89–1.07)	0.91 (0.82–1.02)	0.86 (0.73–1.01)	0.021	0.91 (0.81–1.02)	0.093
Cancer mortality							
Cases	2023	707	430	220	–	–	–
Model 1	Ref.	0.97 (0.89–1.06)	0.99 (0.89–1.10)	1.03 (0.90–1.19)	0.779	1.01 (0.91–1.11)	0.887
Model 2	Ref.	0.98 (0.90–1.07)	1.01 (0.90–1.12)	1.04 (0.90–1.20)	0.604	1.02 (0.92–1.13)	0.660
Low-fat fermented milk	**0 g**	**< 100 g/day**	**100 to < 200 g/day**	**≥ 200 g/day**		**Per 250 g increase**	*P*
All-cause mortality							
Cases/person years	6950/353,522	1305/89,198	947/62,632	540/33,827	–	–	–
Model 1	Ref.	0.88 (0.83–0.94)	0.81 (0.75–0.86)	0.83 (0.76–0.90)	< 0.001	0.82 (0.77–0.88)	< 0.001
Model 2	Ref.	0.93 (0.87–0.98)	0.88 (0.82–0.95)	0.92 (0.84-1.00)	0.001	0.91 (0.85–0.97)	0.006
CVD mortality							
Cases	2221	406	310	178	–	–	–
Model 1	Ref.	0.91 (0.82–1.01)	0.86 (0.76–0.96)	0.87 (0.75–1.01)	0.004	0.85 (0.76–0.95)	0.004
Model 2	Ref.	0.93 (0.84–1.04)	0.94 (0.83–1.06)	0.95 (0.81–1.11)	0.267	0.93 (0.83–1.04)	0.206
Cancer mortality							
Cases	2398	473	329	180	–	–	–
Model 1	Ref.	0.89 (0.80–0.98)	0.81 (0.72–0.91)	0.80 (0.68–0.93)	< 0.001	0.81 (0.72–0.90)	< 0.001
Model 2	Ref.	0.95 (0.86–1.05)	0.90 (0.80–1.01)	0.90 (0.77–1.05)	0.047	0.91 (0.81–1.02)	0.096

^a^Model 1: adjusted for age, sex, dietary assessment version (method), season

Model 2: model 1 plus educational level, leisure-time physical activity, smoking status, alcohol consumption, heredity score of cancer or CVD, marital status, whether living alone, total energy intake, BMI, diet quality index, hypertension, diabetes, lipid-lowering medication, and mutual adjustment between high-fat and low-fat fermented dairy

The association between high-fat cheese and all-cause mortality were similar across strata of stratified variables (Supplementary Table 1). The inverse association between low-fat cheese and all-cause mortality was more pronounced in individuals aged 60 years or older, current and former smokers, or those with lower diet quality (P_interaction_ < 0.05). Low-fat fermented milk also showed a stronger association in current and former smokers.

Our results remained robust in a series of sensitivity analyses, including the exclusion of diet changers, deaths occurring within the first 5 years of follow-up, incident cases of CVD, cancer, or diabetes within the first 5 years of follow-up, energy misreporters, or restricting our study sample to those who reported not altering their diet during the five-year follow-up examination (Supplementary Table 2).

### Fermented dairy intake, plasma proteins, and all-cause mortality risk

Of the 136 proteins, a total of 42, 26, and 39 proteins were identified for the signature of high-fat cheese, low-fat cheese, and low-fat fermented milk intake, respectively. No proteins were identified for high-fat fermented milk intake. Two proteins were shared by all three signatures (Supplementary Fig. 4). The proteomic signatures were significantly correlated with the corresponding fermented dairy intake (*r* = 0.14, 0.13, and 0.17 for high-fat cheese, low-fat cheese, and low-fat fermented milk, respectively; all *P* < 0.001).

**Fig. 3:**
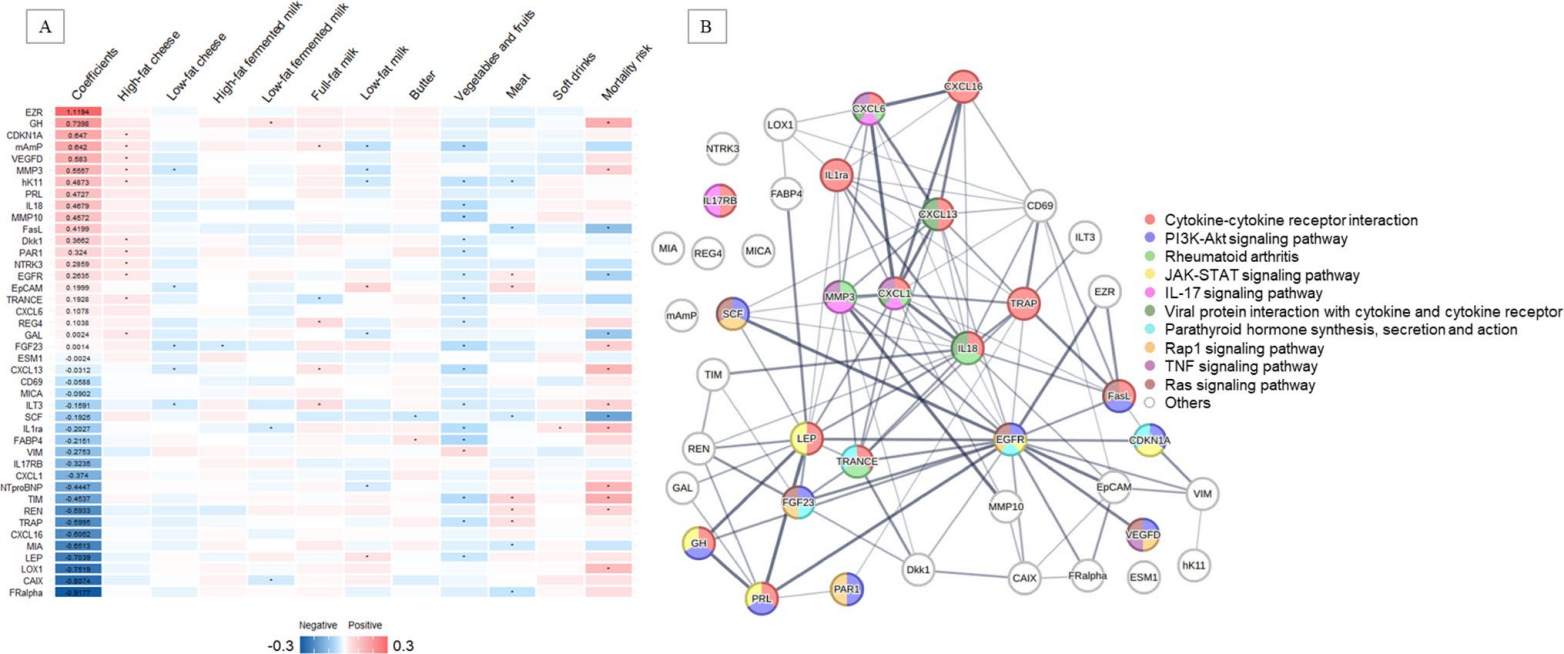
**A** Heatmap showing partial correlations of 42 proteins constituting the proteomic signature of high-fat cheese with other foods and all-cause mortality risk. The first column shows the proteins’ coefficients in the signature of high-fat cheese. * *P* < 0.05. For mortality risk, β (i.e., ln (HR)) was presented. **B** Protein–protein interaction network of 42 proteins. The thickness of the edges reflects the strength of data support. Identified pathways were arranged from top to bottom in ascending order of FDR-adjusted* P* values. Only the top ten pathways are presented

In the fully adjusted models, the HRs (95% CI) for the highest versus lowest quartile were 0.71 (0.60–0.83) for the high-fat cheese signature, 0.75 (0.64–0.88) for the low-fat cheese signature, and 0.64 (0.54–0.76) for the low-fat fermented milk signature (Table 3). Different foods tend to exhibit distinct correlation patterns with proteins (Fig. 3A, Supplementary Fig. 5A, and Supplementary Fig. 6A). Furthermore, the heatmaps showed a pattern where proteins negatively correlated with fermented dairy were generally associated with a higher risk of mortality.

**Table 3:** Hazard ratios (95% CIs) for all-cause mortality by proteomic signatures of fermented dairy^a^

	Categories of proteomic signatures score (quartile)				*P*_trend_	Continuous	
	Q1	Q2	Q3	Q4		Per SD increase	*P*
High-fat cheese							
Cases/person years	539/23,661	421/24,972	365/25,373	269/26,053	–	–	
Model 1	Ref.	0.79 (0.70–0.90)	0.84 (0.73–0.96)	0.66 (0.56–0.77)	< 0.001	0.85 (0.81–0.90)	< 0.001
Model 2	Ref.	0.83 (0.73–0.95)	0.88 (0.77–1.01)	0.71 (0.60–0.83)	< 0.001	0.88 (0.83–0.92)	< 0.001
Low-fat cheese							
Cases/person years	526/23,276	400/24,859	348/25,750	320/26,175	–	–	
Model 1	Ref.	0.78 (0.68–0.89)	0.75 (0.64–0.86)	0.75 (0.64–0.88)	< 0.001	0.88 (0.83–0.93)	< 0.001
Model 2	Ref.	0.79 (0.69–0.90)	0.75 (0.65–0.87)	0.75 (0.64–0.88)	< 0.001	0.88 (0.83–0.93)	< 0.001
Low-fat fermented milk							
Cases/person years	558/23,027	402/25,171	352/25,627	282/26,233	–	–	
Model 1	Ref.	0.78 (0.68–0.89)	0.74 (0.64–0.86)	0.66 (0.56–0.78)	< 0.001	0.84 (0.79–0.89)	< 0.001
Model 2	Ref.	0.76 (0.67–0.87)	0.71 (0.61–0.83)	0.64 (0.54–0.76)	< 0.001	0.82 (0.78–0.87)	< 0.001

^a^Model 1: adjusted for age, sex, season, educational level, leisure-time physical activity, smoking status, alcohol consumption, heredity score of cancer and CVD, total energy intake, marital status, and whether living alone

Model 2: model 1 plus diet quality index, BMI, hypertension, diabetes, and lipid-lowering medication

Although high-fat cheese signature captured 42 proteins, the correlation of each protein with high-fat cheese was generally weak (Fig. 3A). The pathways identified for high-fat cheese and low-fat fermented milk intake were primarily related to immune responses and inflammation, with the cytokine-cytokine receptor interaction being the top pathway (Fig. 3B and Supplementary Fig. 5B). The protective effect of low-fat fermented milk was largely attributed to proteins that were negatively correlated with low-fat fermented milk but were also linked to higher all-cause mortality; this included PRSS8, CTSD, MMP1, IL-8, RETN, HE4, FUR, CCL3, ILT3, MMP12, IL1ra (Supplementary Fig. 5A). IL8 and CCL3 were central players in the network of proteins within the low-fat fermented milk signature (Supplementary Fig. 5B). The rheumatoid arthritis was the top pathway enriched with proteins in the low-fat cheese signature (Supplementary Fig. 6). IL-6 appeared to be a central node within the protein network associated with low-fat cheese intake.

## Discussion

Consumption of high-fat cheese, low-fat cheese, and low-fat fermented milk showed inverse associations with all-cause mortality. Higher high-fat cheese consumption was associated with a lower CVD mortality risk. Mirroring dietary intake, proteomic signatures of high-fat cheese, low-fat cheese, and low-fat fermented milk were inversely associated with all-cause mortality risk. The identified proteins were involved in biological pathways related to immune response and inflammation.

### Fermented dairy intake and mortality risk

Consistent with our findings, a recent meta-analysis reported a lower risk of all-cause mortality (highest vs. lowest: RR = 0.95; 95% CI: 0.92, 0.99) and CVD mortality (RR = 0.93; 95% CI: 0.88, 0.99) with higher cheese intake, but found no association with cancer mortality [6]. In this meta-analysis, fat content of cheese could not be differentiated due to the lack of data. Both low- and high-fat cheese demonstrated an inverse association with all-cause mortality in our study, but the association was stronger for high-fat cheese. Although regular-fat cheese contains a high amount of saturated fatty acids, previous RCTs suggest that it had no more detrimental effects on blood lipid profiles compared to low-fat cheese [33–35]. In porcine models, regular-fat cheese resulted in higher fecal fat and energy excretion, as well as more favorable alterations in gut microbiota, compared to reduced-fat cheese [36]. Alternatively, the weaker association for low-fat cheese may also be due to the relatively low intake levels of low-fat cheese among our participants, as indicated by the significantly wider confidence intervals in the dose-response figure at higher intake levels. Future studies, based on larger cohorts with more participants consuming low-fat cheese, could further validate this.

Our observations on fermented milk were in line with the latest meta-analysis, which reported a 7% lower risk of all-cause mortality and an 11% lower risk of CVD mortality with higher yogurt consumption [7]. We extend previous evidence by considering fat content, suggesting that the reduced all-cause mortality risk associated with fermented milk may be attributed to the low-fat type. Stratified analysis revealed a greater reduction in all-cause mortality risk among smokers. A similar finding was seen in two large US cohorts for yogurt, although the interaction with smoking was not significant [37]. This suggests that low-fat fermented milk might help reduce the mortality risk associated with smoking.

Reverse causation has always been a concern for the findings of low-fat fermented dairy, as some participants might switch from high- to low-fat dairy after a disease diagnosis or during preclinical stages. As observed in our data, participants with a higher intake of low-fat fermented dairy tend to have a higher prevalence of diabetes or use lipid-lowering medication. Nevertheless, when stratified by self-rated health status, results remained consistent across strata. Additionally, excluding deaths within 5 years of baseline, cases of incident CVD, cancer, or diabetes within the first 5 years of follow-up, or restricting the sample to those who reported no significant dietary changes at baseline, still yielded similar results, suggesting a lower likelihood of reverse causation.

### Fermented dairy intake, plasma proteins, and all-cause mortality risk

Alterations in the plasma proteome related to fermented dairy intake primarily involve pathways concerning immune responses and inflammation. The cytokine-cytokine receptor interaction, the top pathway enriched proteins in signatures of high-fat cheese and low-fat fermented milk, was also highlighted as a primary pathway associated with several healthy dietary patterns [38]. A previous study found that a high-fat diet dysregulated the cytokine-cytokine receptor interaction pathway in mouse models [39].

Epidermal growth factor receptor (EGFR), serving as a central player in the network of the high-fat cheese signature, was positively associated with high-fat cheese intake but negatively associated with mortality risk. Its association with mortality risk confirmed by UK Biobank data [40]. EGFR is a membrane-bound protein that plays a role in cell metabolism and adhesion. Higher adherence to several healthy diet patterns has been positively associated with plasma EGFR levels [9, 16]. For low-fat cheese, IL-6 was a central node within the protein network. PRSS8, RETN, HE4, MMP-3, CSF1, ILT3, FGF23, GDF15, and HGF were negatively associated with low-fat cheese, but positively associated with mortality risk, with the associations of PRSS8, RETN, MMP-3, CSF1, FGF23, GDF15, and HGF with mortality risk confirmed in UK Biobank [40]. Human Epididymis Protein 4 (HE4), a secretory protein that functions in immune modulation and epithelial host defense, is recognized as a biomarker for several diseases, including ovarian cancer [41]. MMP-3 activates other matrix metalloproteinases (MMPs) and proinflammatory mediators and is involved in adipose tissue inflammation and fatty acid-induced insulin resistance [42]. Fibroblast growth factor 23 (FGF23) is a hormone that responds to dietary phosphate intake and has been found to be lower with a higher percentage of plant-based protein intake [43]. Hepatocyte growth factor (HGF) has been positively linked to an inflammatory diet [13]. Colony stimulating factor 1 (CSF1) and growth differentiation factor 15 (GDF15) have been negatively associated with multiple healthy dietary patterns and mediated over 50% of the association between Mediterranean diet and all-cause mortality [38].

Higher low-fat fermented milk intake was associated with lower levels of PRSS8, CTSD, MMP-1, IL-8, RETN, FUR, CCL3, MMP-12, and IL-1ra; these proteins were positively associated with all-cause mortality risk, with PRSS8, CTSD, RETN, CCL3, and MMP12 were validated by the UK Biobank study [40]. MMP-12 degrades extracellular matrix components and is involved in tissue remodeling and chronic inflammation [44]. Prostasin (PRSS8) is a serine protease that is crucial for maintaining sodium balance [45]. In an RCT, the Mediterranean diet intervention led to reduced levels of PRSS8 [10]. Cathepsin D (CTSD) is a lysosomal protease involved in protein turnover and extracellular matrix breakdown, and its plasma levels have been positively associated with meat intake [12]. MMP-1 has been found to cleave PAR1 on the surface of platelets, affecting thrombogenesis and atherosclerosis [46]. CCL3, a pro-inflammatory chemokine, is involved in the immune response and has been positively associated with an inflammatory diet [13]. RETN, IL-8, and IL-1ra are pro-inflammatory cytokines. The inverse associations with these inflammatory markers suggest that consuming low-fat fermented milk may reduce inflammation, which could explain its potential survival benefits. Taken together, many proteins in three proteomic signatures have been shown to respond to diet in previous studies, and some of their associations with mortality risk were confirmed in the UK biobank cohort.

### Strengths and limitations

This study is the first to integrate proteomic data to assess the association between fermented dairy intake and mortality risk, and it is also the first to differentiate fat content. Our study was strengthened by its prospective design, long follow-up period, and large sample size. Moreover, the use of a validated diet history method that combined a 7-day food diary helped reduce information bias, while the low rate of loss to follow-up (0.8%) through linkage with national registries minimized attrition bias.

Several limitations should be noted. First, although we adjusted for key confounders, residual confounding due to unmeasured or time-varying risk factors cannot be fully excluded, especially given the long follow-up period of this study. Future studies with repeated assessments of time-varying risk factors may provide further insights. Second, dairy consumption was assessed only once at baseline, which might not reflect long-term intake. However, similar results were found when restricting the study sample to those who reported no significant diet changes at the five-year follow-up examination. Third, we cannot classify high-fat cheese more precisely when the fat content exceeds 20%. Further research is needed to determine whether cheese with higher fat content continues to be associated with a lower risk of death. Fourth, given the great diversity and heterogeneity in nutrients of different cheeses, the lack of data on specific types of cheese in this study limits our ability to provide more precise dietary recommendations. Fifth, the associations between dairy intake and plasma proteins were cross-sectional, which may not indicate causal relationships. Further validation of these associations in RCTs is necessary. Sixth, nonlinear associations between proteins and fermented dairy intake may be missed, as the elastic net regression model assumes linear associations; more advanced machine-learning methods are expected to solve this. Finally, since this study included only the Swedish population, generalizing these findings to other populations warrants caution, considering the variations in background diet (which modified the association between low-fat cheese and mortality risk) and types of commonly consumed cheese.

In conclusion, our data indicated that higher intake of high-fat cheese, low-fat cheese, and low-fat fermented milk was associated with a lower risk of all-cause mortality, while high-fat fermented milk showed no association; proteomic data further strengthened these findings. High-fat cheese intake was also inversely associated with CVD mortality risk. Future studies that differentiate fat content and integrate other omics approaches (e.g., metabolomics) are needed for a comprehensive assessment of the health effects of fermented dairy, particularly high-fat cheese. Functional studies are also warranted to validate and elucidate our results.

## Supplementary Information

Below is the link to the electronic supplementary material.

Supplementary Material 1

## Data Availability

Supporting data are available from the corresponding author upon reasonable request but access to data must be granted by the MDC steering committees.

## Abbreviations

BMI: Body mass index
CVD: Cardiovascular disease
FFQ: Food frequency questionnaire
HR: Hazard ratio
CI: Confidence interval
MDC: Malmö Diet and Cancer
MET: Metabolic equivalent task
RCTs: Randomized controlled trials
SD: Standard deviation
