# Supplementary material for: High-fat and low-fat fermented milk and cheese intake, proteomic signatures, and risk of all-cause and cause-specific mortality
Source: Eur J Nutr. 2025 Oct 15;64(7):297. doi: 10.1007/s00394-025-03815-6 (PMC12528259; doi:10.1007/s00394-025-03815-6)
Supplement: Supplementary file 1 — Supplementary Material 1 [file 394_2025_3815_MOESM1_ESM.docx]

**High-fat and low-fat fermented milk and cheese intake, proteomic signatures, and risk of all-cause and cause-specific mortality**

**Supplemental Content:**

**Supplementary Table 1** Adjusted hazard ratios (95% CIs) for all-cause mortality by daily fermented dairy intake, stratified by key variables.

**Supplementary Table 2** Hazard ratios (95% CIs) for all-cause mortality by fermented dairy intake (**Sensitivity analysis**).

**Supplementary Fig. 1** The flowchart for selecting participants.

**Supplementary Fig. 2** Correlation coefficients between foods and nutrients.

**Supplementary Fig. 3** Distribution of cheese (A) and fermented milk (B) intake by fat content.

**Supplementary Fig. 4** The Venn diagram shows the overlap of proteins in signatures of fermented dairy.

**Supplementary Fig. 5** (A) Heatmap showing partial correlations of 39 proteins constituting the proteomic signature of low-fat fermented milk with other foods and mortality risk. (B) Protein–protein interaction network of 39 proteins.

**Supplementary Fig. 6** (A) Heatmap showing partial correlations of 26 proteins constituting the proteomic signature of low-fat cheese with other foods and mortality risk. (B) Protein–protein interaction network of 26 proteins.

| **Supplementary Table 1** Adjusted hazard ratios (95% CIs) for all-cause mortality by daily fermented dairy intake, stratified by key variables ^a^ | | | | | | | | | | | |
| --- | --- | --- | --- | --- | --- | --- | --- | --- | --- | --- | --- |
|  | **High-fat cheese** | |  | **Low-fat cheese** | |  | **High-fat fermented milk** | |  | **Low-fat fermented milk** | |
|  | Per 20 g increase | P_interaction_ |  | Per 20 g increase | P_interaction_ |  | Per 250 g increase | P_interaction_ |  | Per 250 g increase | P_interaction_ |
| **Sex** |  |  |  |  |  |  |  |  |  |  |  |
| Males | 0.97 (0.95-0.99) | 0.742 |  | 0.97 (0.93-1.00) | 0.244 |  | 0.94 (0.86-1.02) | 0.076 |  | 0.92 (0.84-1.01) | 0.654 |
| Females | 0.98 (0.96-1.00) |  |  | 0.99 (0.96-1.02) |  |  | 1.04 (0.95-1.14) |  |  | 0.92 (0.84-1.01) |  |
| **Age at recruitment** |  |  |  |  |  |  |  |  |  |  |  |
| < 60 years | 0.95 (0.93-0.98) | 0.107 |  | **0.99 (0.96-1.02)** | **0.020** |  | 1.04 (0.94-1.15) | 0.373 |  | 0.85 (0.76-0.95) | 0.148 |
| ≥ 60 years | 0.97 (0.95-1.00) |  |  | **0.96 (0.93-0.98)** |  |  | 0.98 (0.91-1.06) |  |  | 0.92 (0.85-0.99) |  |
| **Body mass index** |  |  |  |  |  |  |  |  |  |  |  |
| < 25 kg/m^2^ | 0.97 (0.95-0.99) | 0.877 |  | 0.96 (0.92-0.99) | 0.267 |  | 0.98 (0.90-1.08) | 0.970 |  | 0.90 (0.81-0.99) | 0.301 |
| ≥ 25 kg/m^2^ | 0.97 (0.95-1.00) |  |  | 0.99 (0.97-1.02) |  |  | 0.97 (0.89-1.05) |  |  | 0.94 (0.86-1.02) |  |
| **Smoking status** |  |  |  |  |  |  |  |  |  |  |  |
| Current or former | 0.96 (0.94-0.98) | 0.965 |  | **0.96 (0.93-0.98)** | **0.021** |  | 0.93 (0.86-1.01) | 0.087 |  | **0.85 (0.78-0.92)** | **0.018** |
| Never | 0.98 (0.95-1.01) |  |  | **1.01 (0.97-1.05)** |  |  | 1.06 (0.96-1.17) |  |  | **0.95 (0.86-1.06)** |  |
| **Drinking status** |  |  |  |  |  |  |  |  |  |  |  |
| < median | 0.98 (0.96-1.00) | 0.953 |  | 0.99 (0.96-1.02) | 0.398 |  | 1.01 (0.94-1.09) | 0.181 |  | 0.94 (0.87-1.02) | 0.082 |
| ≥ median | 0.96 (0.93-0.98) |  |  | 0.96 (0.92-1.00) |  |  | 0.90 (0.81-1.01) |  |  | 0.88 (0.78-0.98) |  |
| **Physical activity** |  |  |  |  |  |  |  |  |  |  |  |
| < 25 METh/week | 0.97 (0.95-0.99) | 0.489 |  | 0.96 (0.93-0.99) | 0.069 |  | 1.02 (0.93-1.11) | 0.844 |  | 0.92 (0.83-1.01) | 0.840 |
| ≥ 25 METh/week | 0.97 (0.95-0.99) |  |  | 0.99 (0.96-1.02) |  |  | 0.95 (0.88-1.03) |  |  | 0.91 (0.84-1.00) |  |
| **Diet sore index** |  |  |  |  |  |  |  |  |  |  |  |
| Low | 0.97 (0.95-0.99) | 0.999 |  | **0.94 (0.90-0.98)** | **0.047** |  | 0.96 (0.88-1.06) | 0.376 |  | 0.87 (0.78-0.97) | 0.377 |
| High | 0.97 (0.95-0.99) |  |  | **0.99 (0.96-1.01)** |  |  | 0.99 (0.92-1.07) |  |  | 0.94 (0.87-1.01) |  |
| **Self-rated health condition** |  |  |  |  |  |  |  |  |  |  |  |
| Poor | 0.97 (0.95-0.99) | 0.346 |  | 0.98 (0.95-1.01) | 0.512 |  | 1.04 (0.96-1.13) | 0.065 |  | 0.88 (0.80-0.96) | 0.120 |
| Good | 0.97 (0.95-1.00) |  |  | 0.98 (0.94-1.01) |  |  | 0.92 (0.84-1.01) |  |  | 0.96 (0.87-1.05) |  |
| ^a^ The variable categories used in the stratified analysis are as follows: alcohol consumption (sex-specific median: < median/≥median), diet quality (<2/≥2), self-rated health condition (<6/≥6). Covariates in model 2 were adjusted. | | | | | | | | | | | |

| **Supplementary Table 2** Hazard ratios (95% CIs) for all-cause mortality by fermented dairy intake (**Sensitivity analysis**) ^a^ | | | | | | | |
| --- | --- | --- | --- | --- | --- | --- | --- |
| **High-fat cheese** | **< 15 g/day** | **15- 30 g/day** | **30 to < 50 g/day** | **≥ 50 g/day** | **P_trend_** | **Per 20 g increase** | **P** |
| Excluding diet changers | 1.00 | 0.98 (0.92-1.05) | 0.91 (0.85-0.98) | 0.91 (0.85-0.98) | 0.004 | 0.98 (0.96-1.00) | 0.017 |
| Excluding energy misreporters | 1.00 | 0.97 (0.91-1.03) | 0.92 (0.87-0.99) | 0.90 (0.84-0.97) | 0.002 | 0.97 (0.95-0.99) | 0.001 |
| No substantial change in diet during the 5-year follow-up examination | 1.00 | 0.96 (0.90-1.03) | 0.92 (0.85-0.98) | 0.91 (0.84-0.99) | 0.013 | 0.98 (0.96-1.00) | 0.047 |
| Excluding CVD, diabetes, cancer occuring within 5 years after recuritment | 1.00 | 0.98 (0.92-1.04) | 0.94 (0.88-1.00) | 0.91 (0.85-0.97) | 0.004 | 0.97 (0.95-0.99) | 0.001 |
| Excluding deaths occuring within 5 years after recuritment | 1.00 | 0.97 (0.92-1.03) | 0.92 (0.87-0.98) | 0.91 (0.85-0.97) | 0.002 | 0.97 (0.96-0.99) | 0.001 |
| **Low-fat cheese** | **0 g/day** | **< 10 g/day** | **10 to < 30 g/day** | **≥ 30 g/day** | **P_trend_** | **Per 20 g increase** | **P** |
| Excluding diet changers | 1.00 | 0.97 (0.91-1.04) | 0.95 (0.88-1.02) | 0.89 (0.81-0.97) | 0.007 | 0.97 (0.94-0.99) | 0.021 |
| Excluding energy misreporters | 1.00 | 0.97 (0.91-1.04) | 0.95 (0.88-1.02) | 0.97 (0.90-1.05) | 0.351 | 0.98 (0.96-1.01) | 0.147 |
| No substantial change in their diet during the 5-year follow-up examination | 1.00 | 0.91 (0.85-0.98) | 0.89 (0.82-0.97) | 0.98 (0.90-1.07) | 0.468 | 0.99 (0.96-1.01) | 0.332 |
| Excluding CVD, diabetes, cancer occuring within 5 years after recuritment | 1.00 | 0.95 (0.89-1.01) | 0.91 (0.85-0.97) | 0.98 (0.91-1.06) | 0.376 | 0.98 (0.96-1.01) | 0.150 |
| Excluding deaths occuring within 5 years after recuritment | 1.00 | 0.96 (0.90-1.02) | 0.92 (0.86-0.98) | 0.97 (0.90-1.04) | 0.194 | 0.98 (0.96-1.00) | 0.063 |
| **High-fat fermented milk** | **0 g/day** | **< 100 g/day** | **100 to < 200 g/day** | **≥ 200 g/day** | **P_trend_** | **Per 250 g increase** | **P** |
| Excluding diet changers | 1.00 | 0.98 (0.92-1.04) | 0.97 (0.91-1.05) | 0.97 (0.88-1.07) | 0.385 | 0.98 (0.91-1.05) | 0.581 |
| Excluding energy misreporters | 1.00 | 0.97 (0.92-1.03) | 0.96 (0.90-1.03) | 0.97 (0.89-1.07) | 0.296 | 0.97 (0.91-1.04) | 0.442 |
| No substantial change in their diet during the 5-year follow-up examination | 1.00 | 0.99 (0.92-1.05) | 1.01 (0.94-1.09) | 0.99 (0.89-1.10) | 0.961 | 1.00 (0.93-1.08) | 0.984 |
| Excluding CVD, diabetes, cancer occuring within 5 years after recuritment | 1.00 | 0.96 (0.91-1.02) | 0.97 (0.91-1.04) | 0.98 (0.89-1.08) | 0.503 | 0.99 (0.93-1.06) | 0.782 |
| Excluding deaths occuring within 5 years after recuritment | 1.00 | 0.97 (0.92-1.02) | 0.97 (0.91-1.03) | 0.97 (0.89-1.06) | 0.273 | 0.98 (0.92-1.04) | 0.527 |
| **Low-fat fermented milk** | **0 g/day** | **< 100 g/day** | **100 to < 200 g/day** | **≥ 200 g/day** | **P_trend_** | **Per 250 g increase** | **P** |
| Excluding diet changers | 1.00 | 0.92 (0.86-0.99) | 0.90 (0.83-0.98) | 0.94 (0.84-1.05) | 0.017 | 0.92 (0.85-1.00) | 0.041 |
| Excluding energy misreporters | 1.00 | 0.94 (0.88-1.00) | 0.90 (0.84-0.97) | 0.95 (0.87-1.05) | 0.026 | 0.93 (0.87-1.00) | 0.050 |
| No substantial change in their diet during the 5-year follow-up examination | 1.00 | 0.92 (0.85-0.99) | 0.92 (0.84-1.00) | 0.94 (0.84-1.05) | 0.050 | 0.93 (0.86-1.00) | 0.064 |
| Excluding CVD, diabetes, cancer occuring within 5 years after recuritment | 1.00 | 0.92 (0.86-0.98) | 0.88 (0.82-0.95) | 0.92 (0.84-1.02) | 0.002 | 0.91 (0.85-0.98) | 0.010 |
| Excluding deaths occuring within 5 years after recuritment | 1.00 | 0.93 (0.87-0.99) | 0.90 (0.83-0.96) | 0.94 (0.85-1.02) | 0.005 | 0.93 (0.87-0.99) | 0.029 |
| ^a^ Covariates in model 2 were adjusted. | | | | | | | |


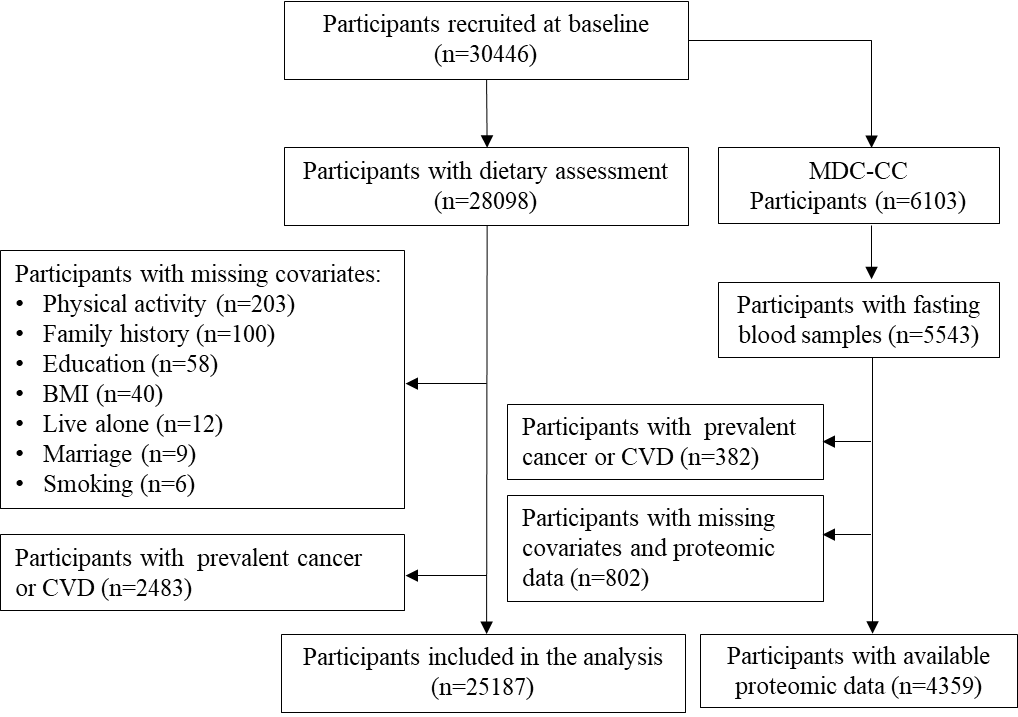


**Supplementary Fig. 1** The flowchart for selecting participants.


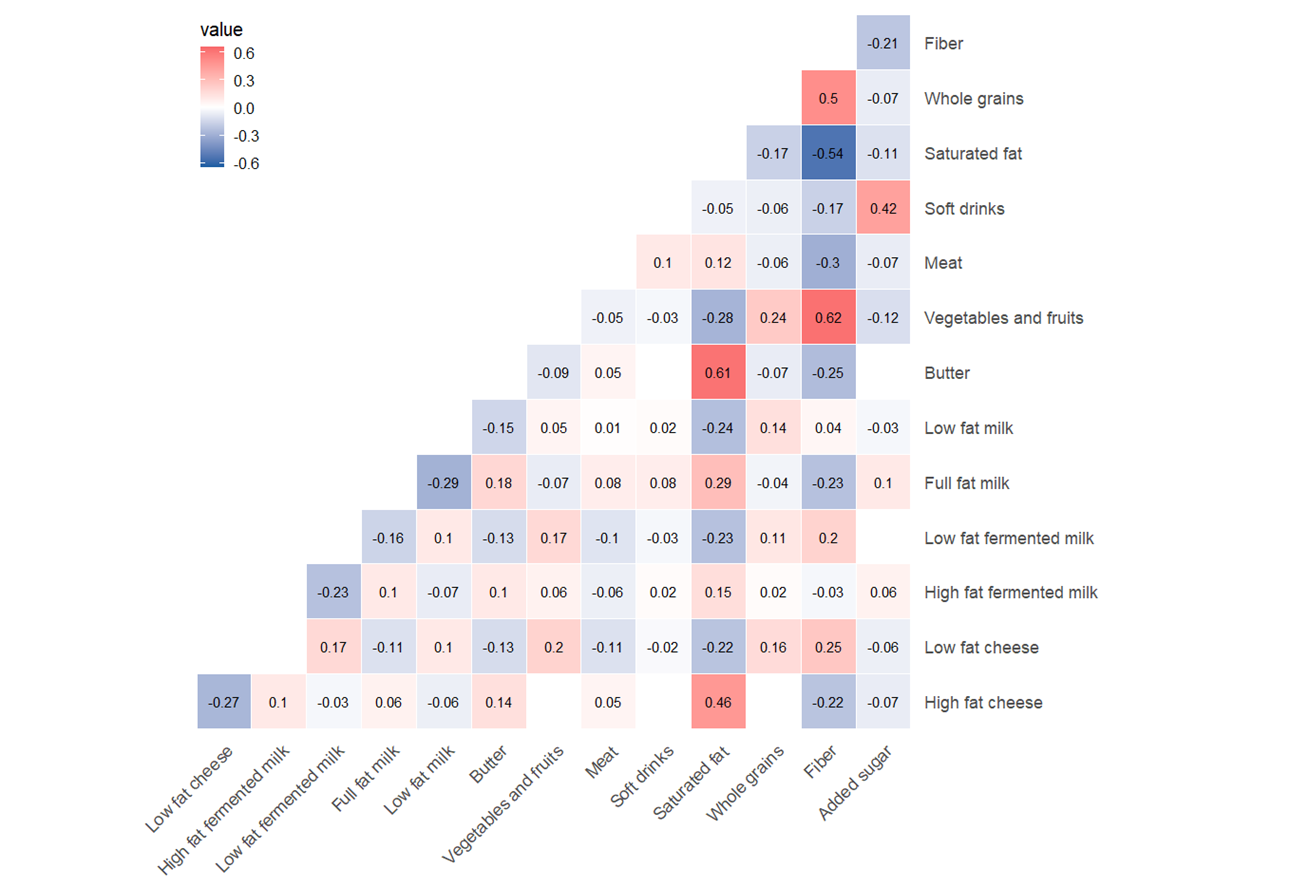


**Supplementary Fig.2** Correlation coefficients between foods and nutrients. Pairs with P value > 0.05 were not shown.


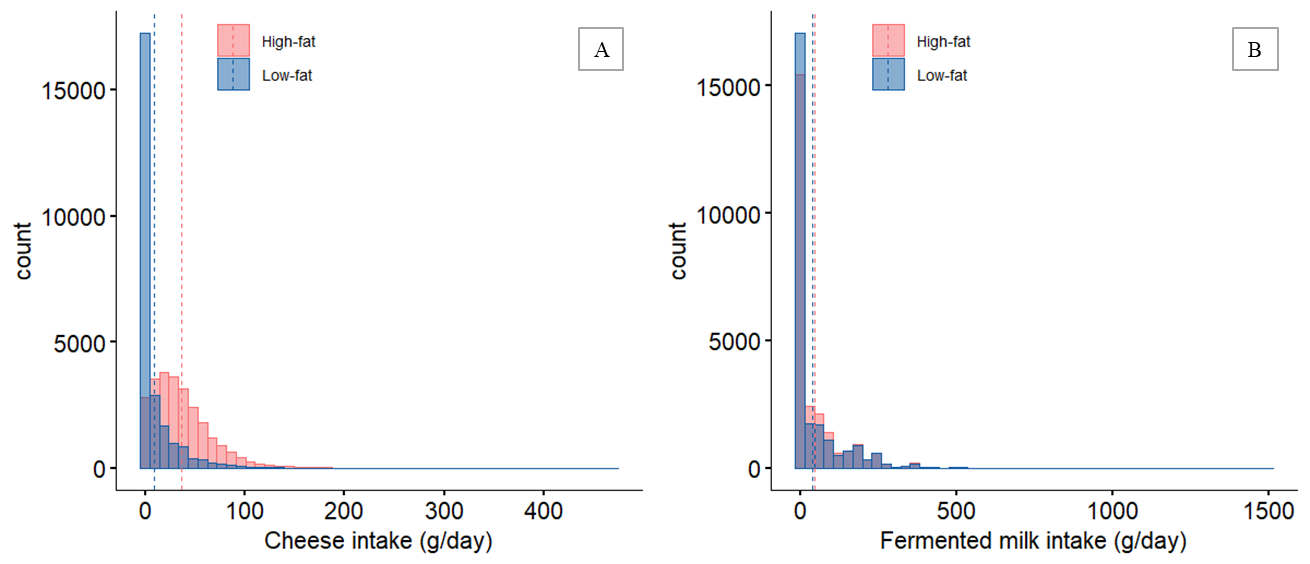


**Supplementary Fig. 3** Distribution of cheese (A) and fermented milk (B) intake by fat content. The dashed lines represent the mean intake.


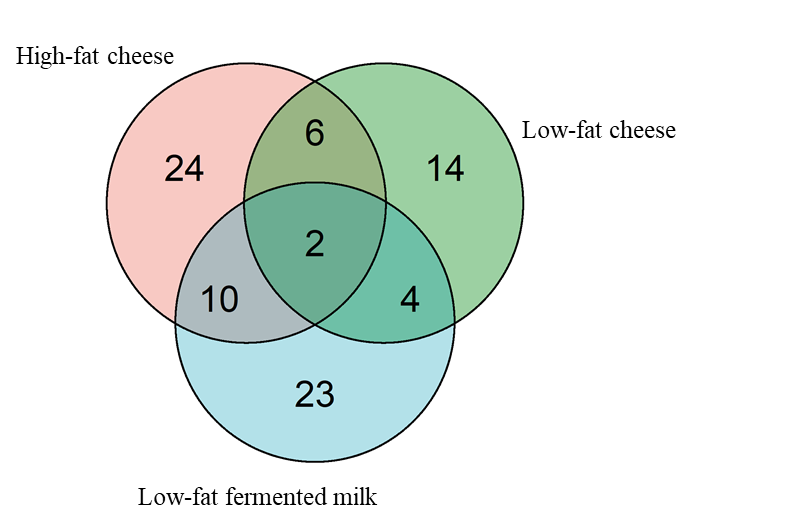


**Supplementary Fig.4** The Venn diagram shows the overlap of proteins in three signatures.


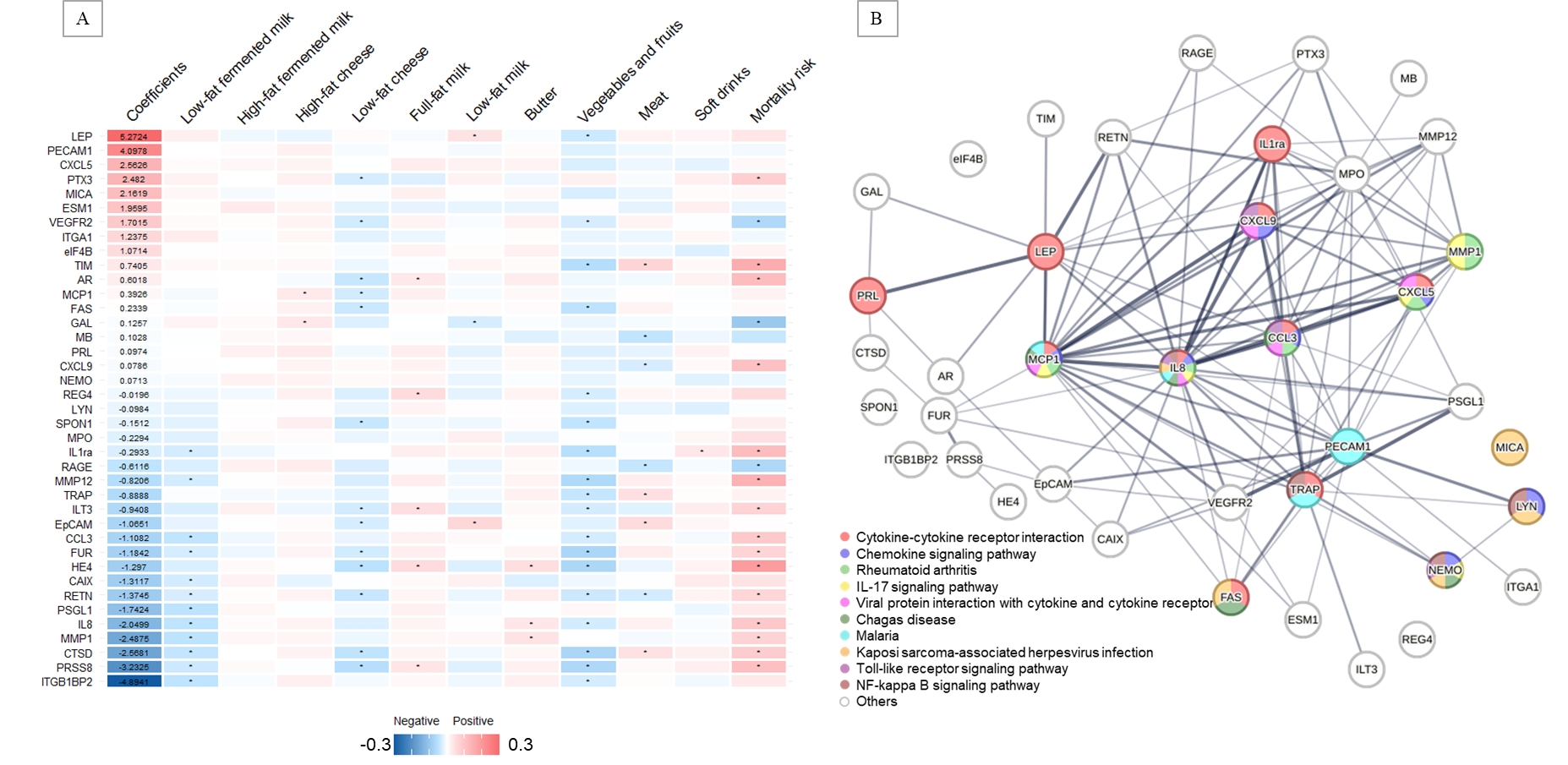


**Supplementary Fig.5** (A) Heatmap showing partial correlations of 39 proteins constituting the proteomic signature of low-fat fermented milk with other foods and mortality risk. The first column shows the proteins' coefficients in the signature of low-fat fermented milk. * P < 0.05. For mortality risk, β (i.e., ln (HR)) was presented. (B) Protein–protein interaction network of 39 proteins. The thickness of the edges reflects the strength of data support. Identified pathways were arranged from top to bottom in ascending order of FDR-adjusted P values. Only the top ten pathways are presented.


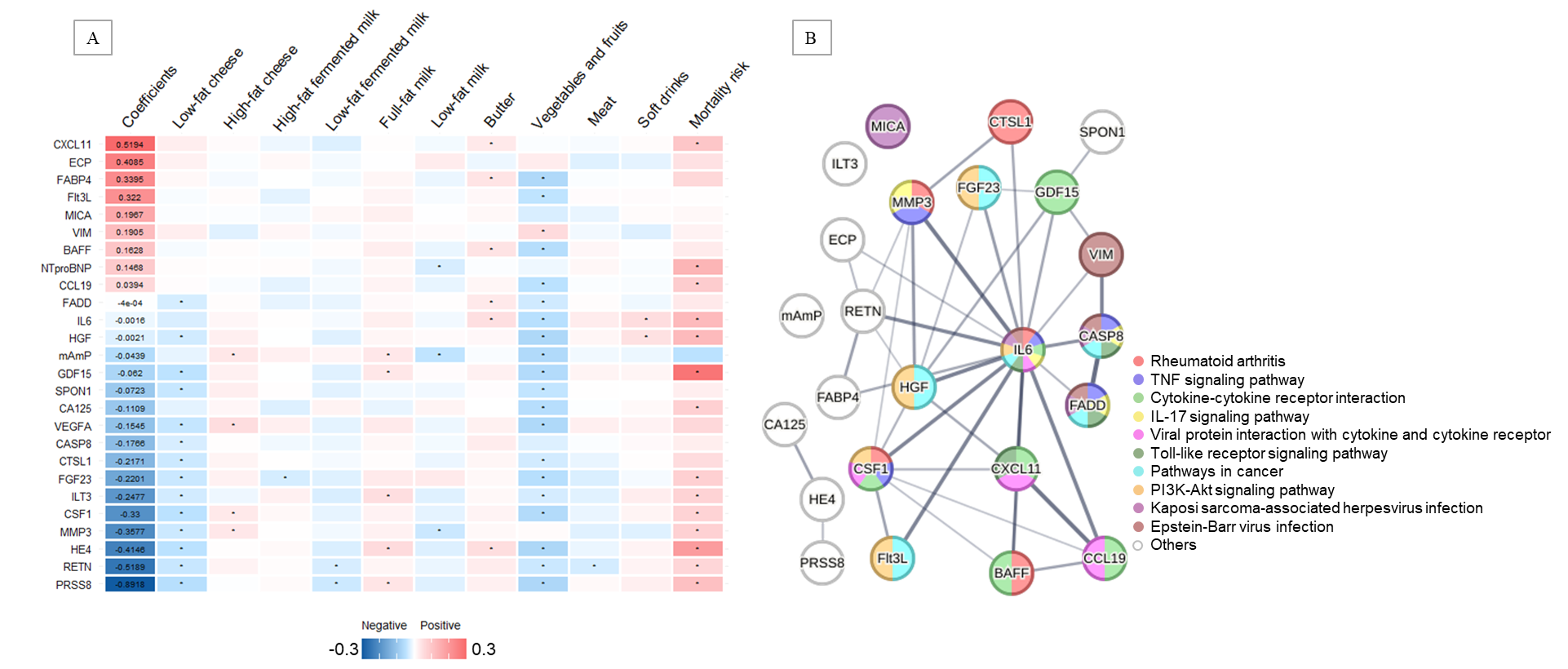


**Supplementary Fig.6** (A) Heatmap showing partial correlations of 26 proteins constituting the proteomic signature of low-fat cheese with other foods and mortality risk. The first column shows the proteins' coefficients in the signature of low-fat cheese. * P < 0.05. For mortality risk, β (i.e., ln (HR)) was presented. (B) Protein–protein interaction network of 26 proteins. The thickness of the edges reflects the strength of data support. Identified pathways were arranged from top to bottom in ascending order of FDR-adjusted P values. Only the top ten pathways are presented. NTproBNP was not presented because it was not found in the STRING database.
